# Supplementary material for: PCR-like performance of rapid test with permselective tunable nanotrap
Source: Nat Commun. 2023 Mar 18;14:1520. doi: 10.1038/s41467-023-37018-6 (PMC10024276; doi:10.1038/s41467-023-37018-6)
Supplement: Supplementary file 1 — Supplementary Information [file 41467_2023_37018_MOESM1_ESM.pdf]

PCR-like Performance of Rapid Test with Permselective Tunable Nanotrap

Seong Jun Park[1,†], Seungmin Lee[1,2,†], Dongtak Lee[2,†], Na Eun Lee[1,3], Jeong Soo Park[1], Ji Hye Hong[1,2], Jae Won Jang[2,4], Hyunji Kim[2,4], Seokbeom Roh[5,6], Gyudo Lee[5,6], Dongho Lee[7], Sung-Yeon Cho[8,9], Chulmin Park[8], Dong-Gun Lee[8,9], Raeseok Lee[8,9], Dukhee Nho[8,9], Dae Sung Yoon[2,4,10,*], Yong Kyoung Yoo[11,*] and Jeong Hoon Lee[1,*]

[1] Department of Electrical Engineering, Kwangwoon University, 20 Kwangwoon-ro, Nowon, Seoul 01897, Republic of Korea

[2] School of Biomedical Engineering, Korea University, 145 Anam-ro, Seongbuk, Seoul 02841, Republic of Korea

[3] Department of Biotechnology, College of Life Sciences and Biotechnology, Korea University, Seoul 02841, Republic of Korea

[4] Interdisciplinary Program in Precision Public Health, Korea University, Seoul 02841, Republic of Korea

[5] Department of Biotechnology and Bioinformatics, Korea University, Sejong 30019, Republic of Korea

[6] Interdisciplinary Graduate Program for Artificial Intelligence Smart Convergence Technology, Korea University, Sejong 30019, Korea

[7] CALTH Inc., Changeop-ro 54, Seongnam, Gyeonggi 13449, Republic of Korea

[8] Vaccine Bio Research Institute, College of Medicine, The Catholic University of Korea, Seoul, Republic of Korea

[9] Division of Infectious Diseases, Department of Internal Medicine, College of Medicine, The Catholic University of Korea, Seoul, Republic of Korea

[10] Astrion Inc, Seoul 02841, Republic of Korea

[11] Department of Electronic Engineering, Catholic Kwandong University, 24, Beomil-ro 579 beon-gil, Gangneung-si, Gangwon-do 25601, Republic of Korea

[†] These authors contributed equally: Seong Jun Park, Seungmin Lee, Dongtak Lee

[*] These authors jointly supervised this work: Dae Sung Yoon, Yong Kyoung Yoo, Jeong Hoon Lee

e-mail: dsyoon@korea.ac.kr; yongkyoung0108@cku.ac.kr; jhlee@kw.ac.kr

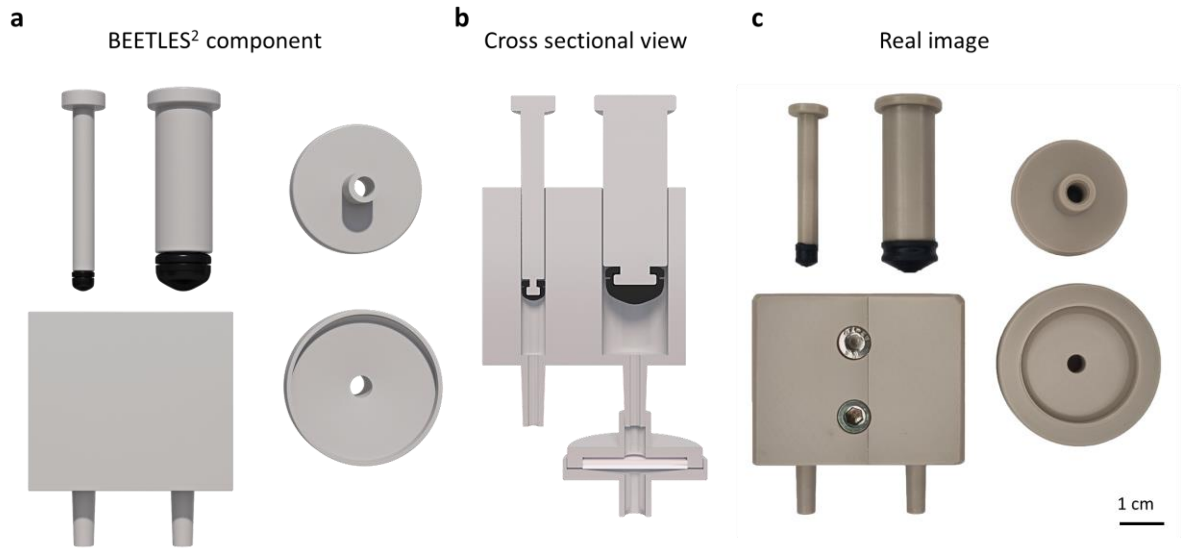

Supplementary Fig. 1. (a–c) Prototype for POCT sample preparation. POCT, point-of-care test.

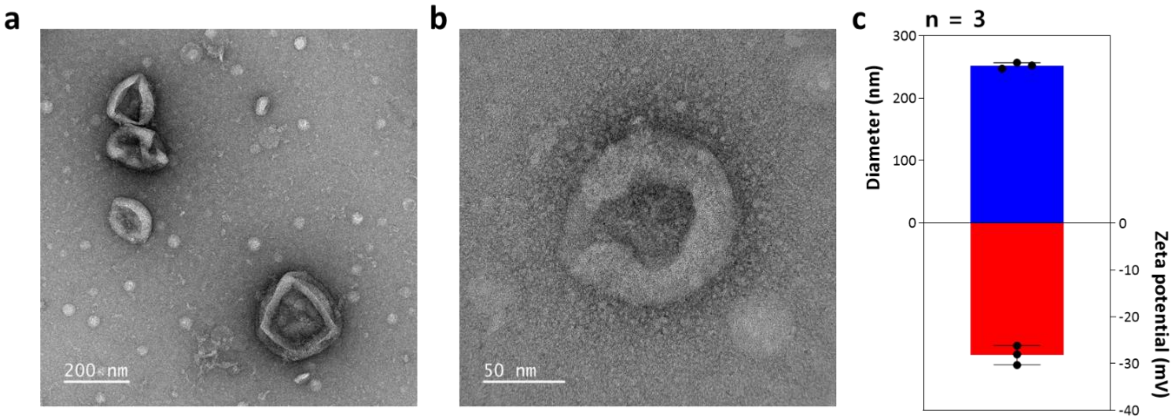

Supplementary Fig. 2. (a–b) Images of the extracted RBCM vesicles, and (c) hydrodynamic size (diameter in nm) and zeta potentials (mV) of the extracted RBCM vesicles. Error bars represent standard deviation from the mean. RBCM, red blood cell membrane.

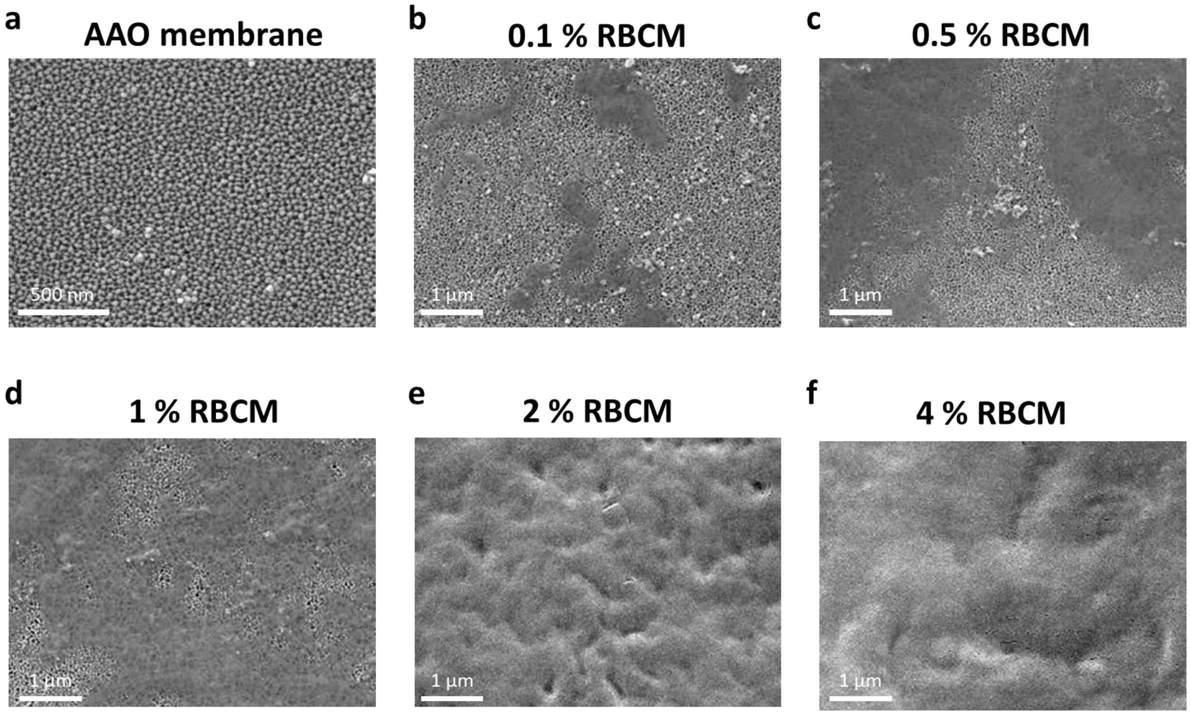

Supplementary Fig. 3. (a–f) SEM images of the bare and BEETLES^2 membrane with various RBCM concentrations (0–4% (v/v)). SEM, scanning electron microscopy; BEETLES^2, bioengineered enrichment tools for the LFA with enhanced sensitivity and specificity; LFA, lateral flow assay; RBCM, red blood cell membrane.

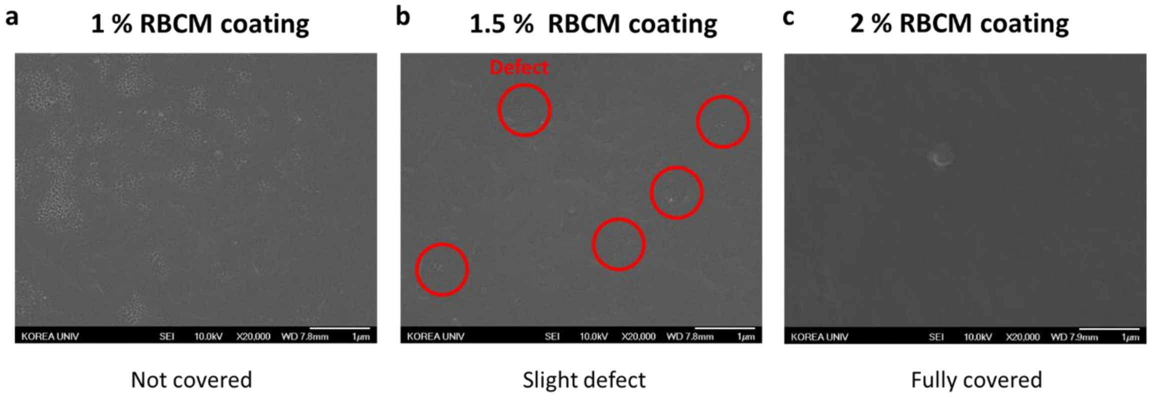

Supplementary Fig. 4. SEM images of the BEETLES^2 membrane surface for optimizing RBCM concentration. (a) 1% RBCM coating, (b) 1.5% RBCM coating, and (c) 2% RBCM coating. SEM, scanning electron microscope; BEETLES^2, bioengineered enrichment tools for the LFA with enhanced sensitivity and specificity; RBCM, red blood cell membrane.

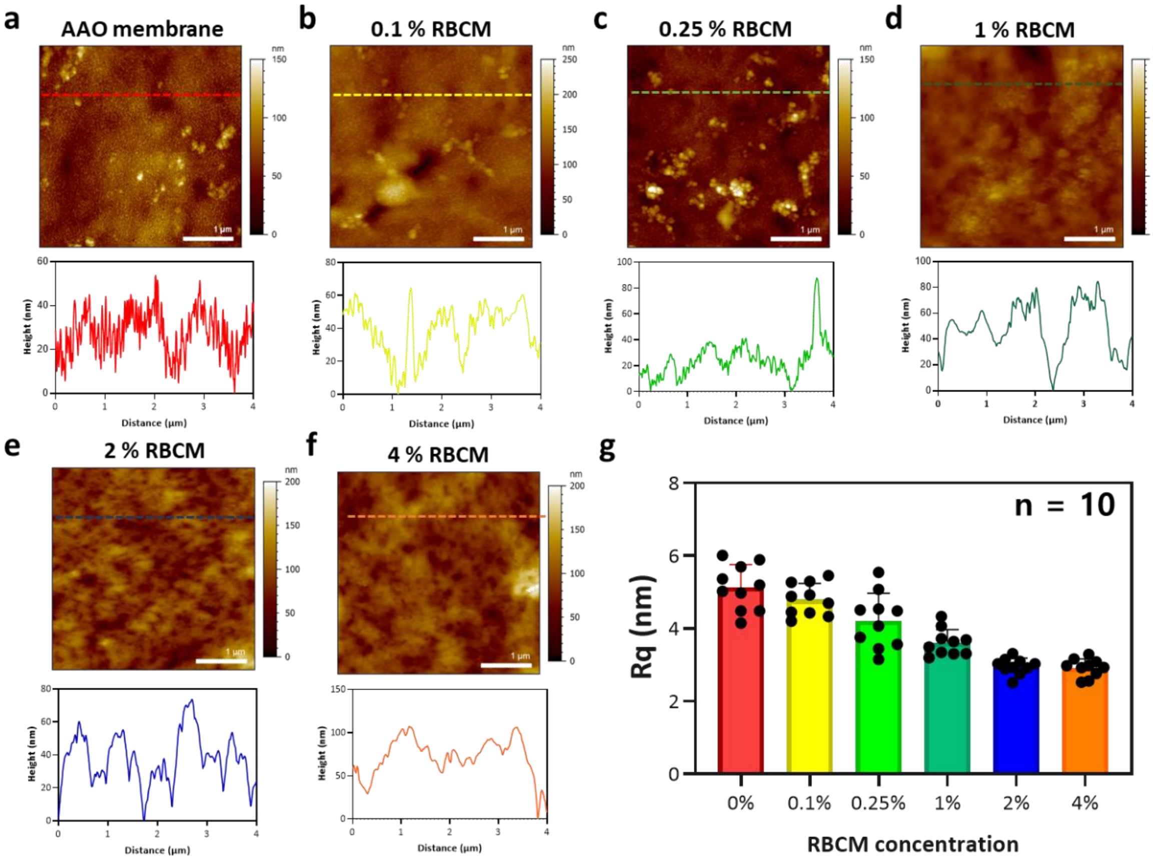

Supplementary Fig. 5. (a–f) Topological images and cross-sectional profiles of the bare AAO and BEETLES^2 membranes with various RBCM concentrations (0–4% (v/v)). (g) Surface roughness analysis with various RBCM concentrations, indicating that 2% RBCM is the optimal concentration for the fabrication of BEETLES^2. Error bars represent standard deviation from the mean. BEETLES^2, bioengineered enrichment tools for the LFA with enhanced sensitivity and specificity; LFA, lateral flow assay; RBCM, red blood cell membrane; AAO, anodic aluminum oxide.

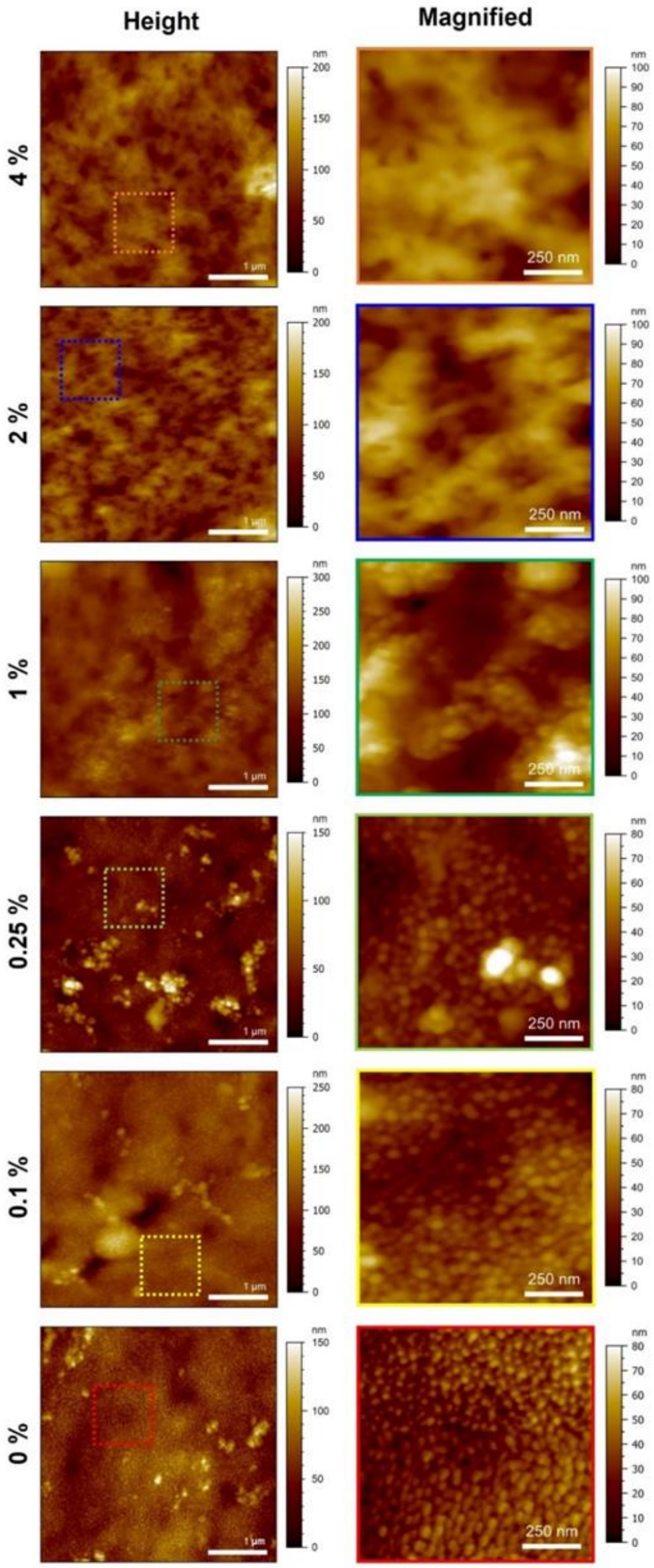

Supplementary Fig. 6. Magnified AFM images of the AAO membrane with different RBCM concentrations. AFM, atomic force microscope; AAO, anodic aluminum oxide; RBCM, red blood cell membrane.

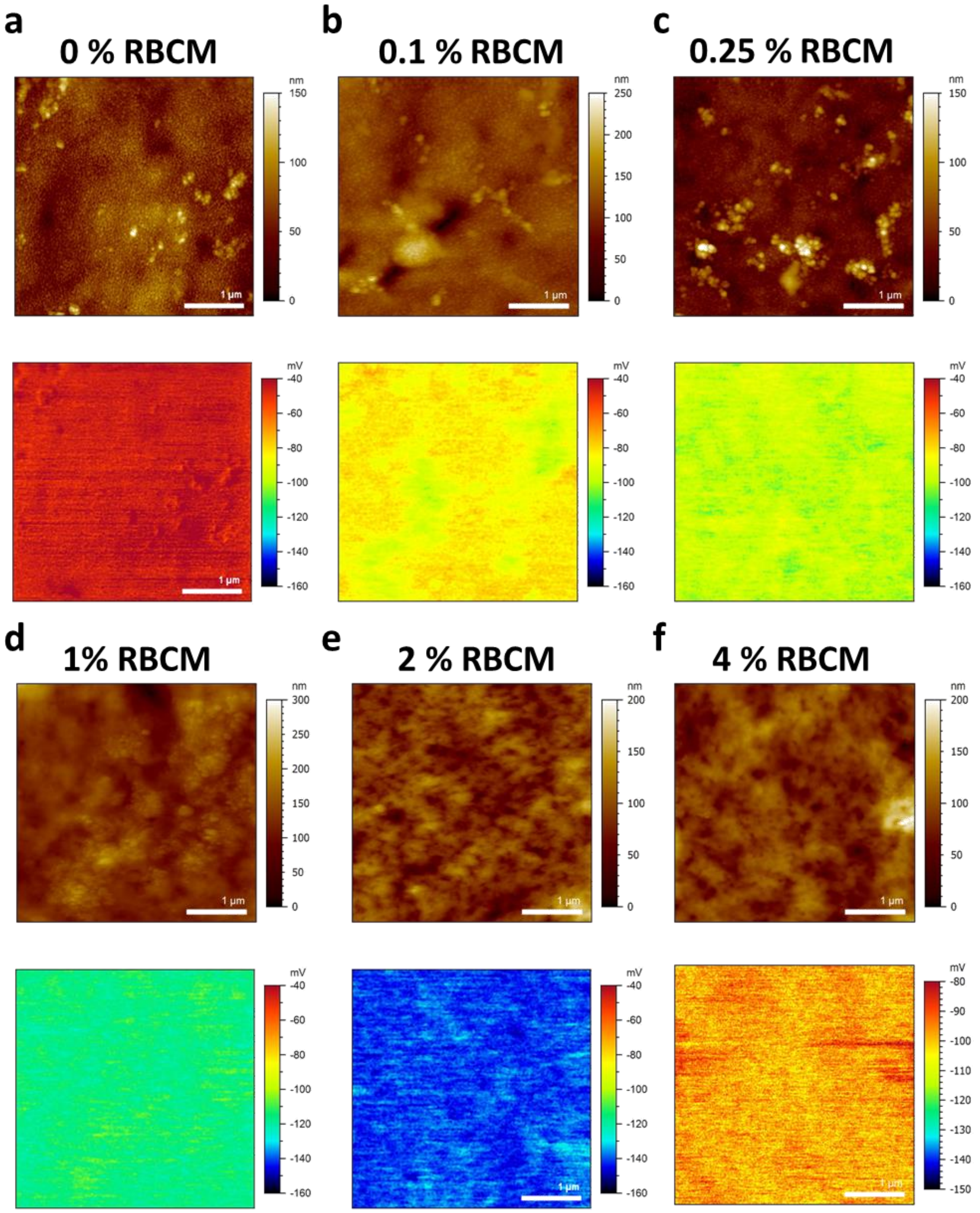

Supplementary Fig. 7. (a–f) Surface potential mapping by KPFM with various RBCM concentrations (0–4% (v/v)), showing that the BEETLES^2 membrane is negatively charged because of the negatively charged phospholipids in RBCM. KPFM, kelvin probe force microscopy; BEETLES^2, bioengineered enrichment tools for the LFA with enhanced sensitivity and specificity; LFA, lateral flow assay; RBCM, red blood cell membrane.

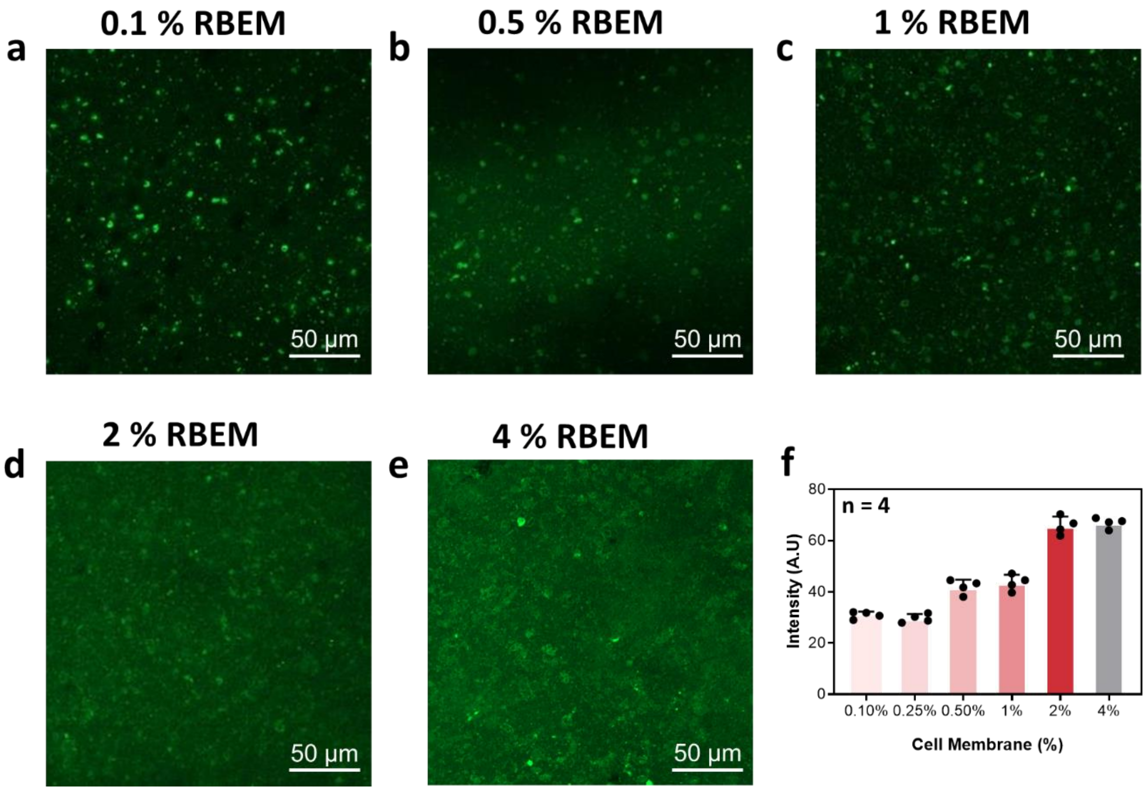

Supplementary Fig. 8. (a–e) Fluorescent images of BEETLES^2 with various RBCM concentrations (0–4% (v/v)). (f) Quantitative analysis of fluorescent intensity depending on various RBCM concentrations, indicating that RBCM deposition is saturated at 2% RBCM. Error bars represent standard deviation from the mean. BEETLES^2, bioengineered enrichment tools for the LFA with enhanced sensitivity and specificity; LFA, lateral flow assay; RBCM, red blood cell membrane.

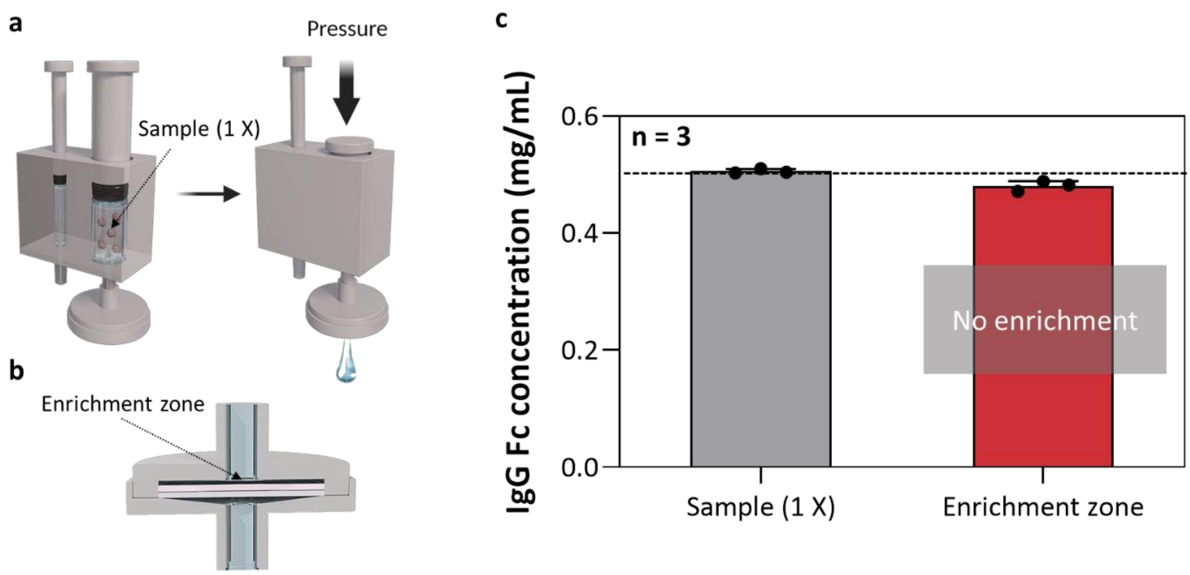

Supplementary Fig. 9. Size effect of IgG on enrichment. (a) Enrichment process with BEETLES[2] (b) Enrichment zone of BEETLES[2] (c) No enrichment from IgG fragment (IgG Fc fragment molecular weight: 50 kDa). Error bars represent standard deviation from the mean. Cartoons in panels a, b were created with BioRender.com. IgG, immunoglobulin G; BEETLES[2], bioengineered enrichment tools for LFA with enhanced sensitivity and specificity.

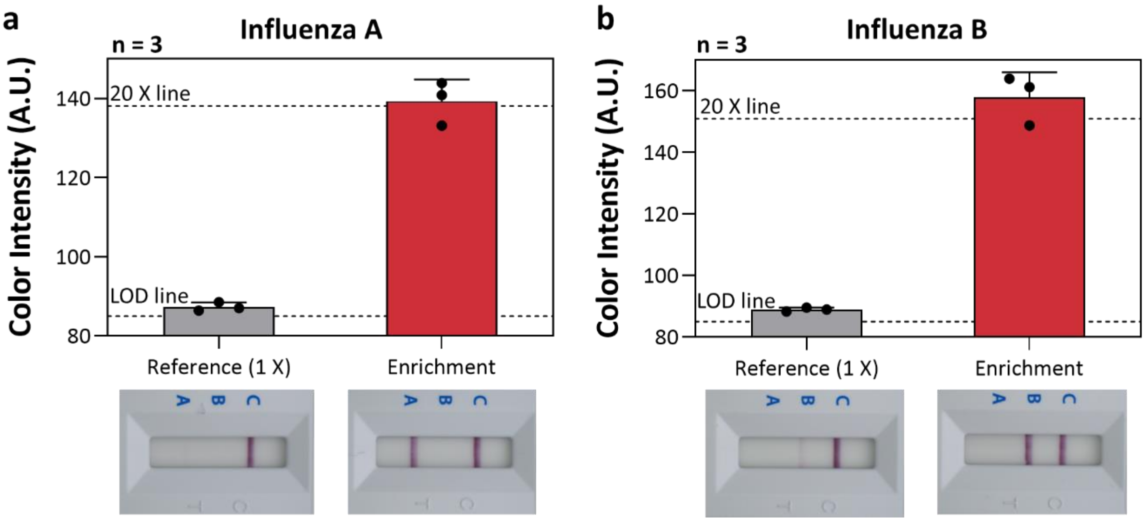

Supplementary Fig. 10. Enhanced sensitivity assay of Influenza A/B commercial kits via BEETLES^2. Color intensity of each assay (top) and LFA kit image (bottom). (a) N protein validation for Influenza A and (b) Influenza B. Error bars represent standard deviation from the mean. BEETLES^2, bioengineered enrichment tools for LFA with enhanced sensitivity and specificity; LFA, lateral flow assay; N protein, nucleocapsid protein.

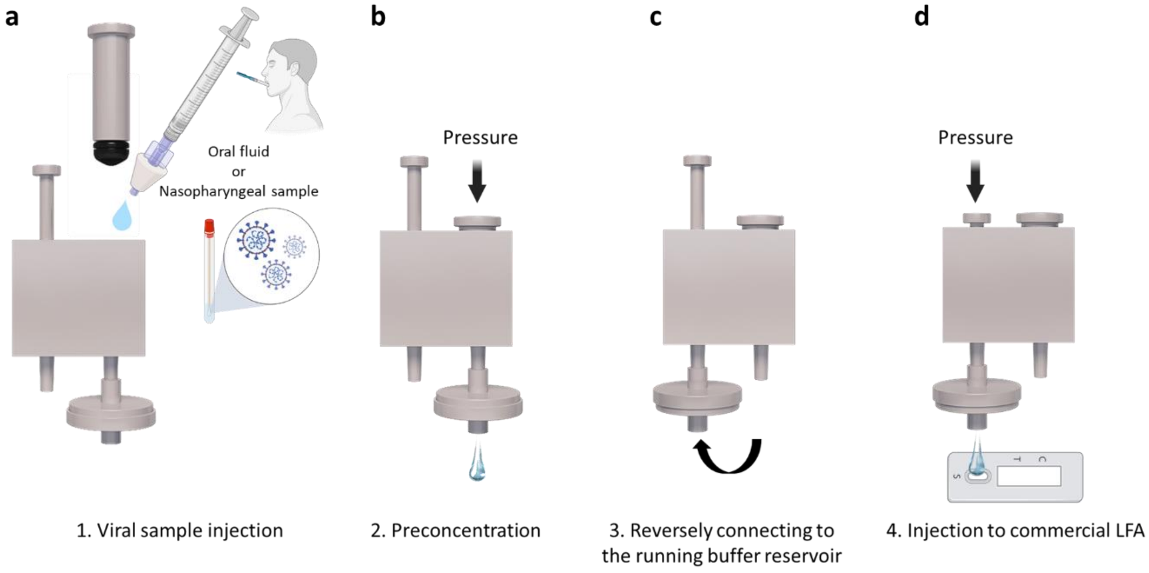

Supplementary Fig. 11. (a–d) Assay process of a hand-powered portable gadget integrated with BEETLES^2. The system contains two reservoirs: sample and commercially available running buffer reservoirs. Cartoons in panels a-d were created with BioRender.com. BEETLES^2, bioengineered enrichment tools for the LFA with enhanced sensitivity and specificity; LFA, lateral flow assay.

a

**Cut-off value** (color intensity (A.U.))	**86**
w/o BEETLES^2 data near cut-off line	**85.992**
w/ BEETLES^2 date near cut-off line	**86.338**

b **c**

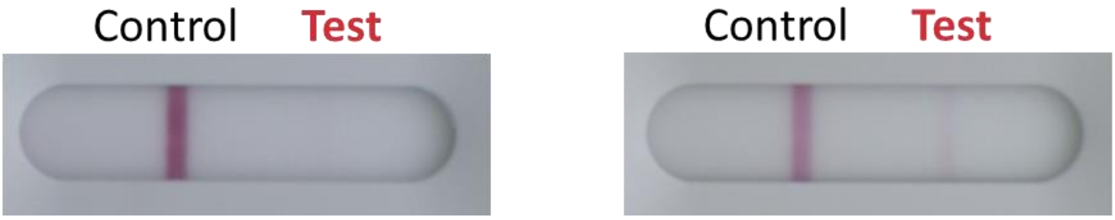

Supplementary Fig. 12. (a) Color intensities, (b) image w/o BEETLES^2 data slightly below the cut-off line, and (c) image w/ BEETLES^2 data slightly above the cut-off line. BEETLES^2, bioengineered enrichment tools for the LFA with enhanced sensitivity and specificity.

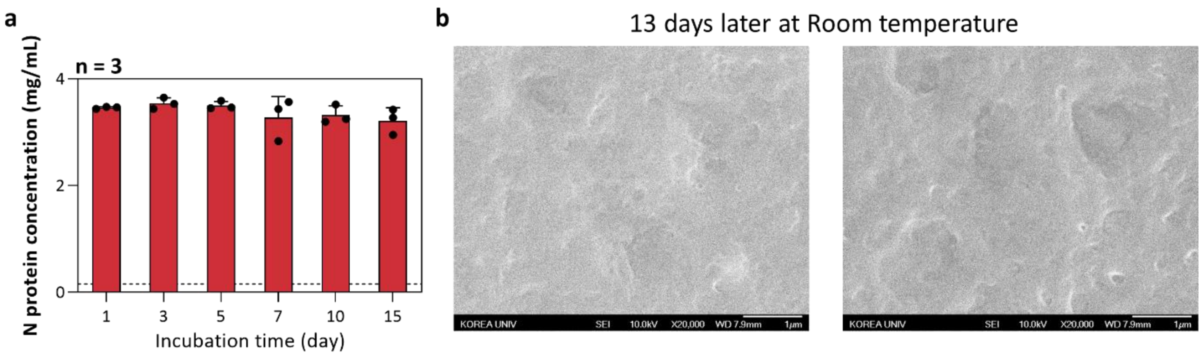

Supplementary Fig. 13. Shelf-life test for commercializing the BEETLES^2 membrane. (a) N protein enrichment performance test of the BEETLES^2 membrane incubated at room temperature. (b) SEM images. Error bars represent standard deviation from the mean. BEETLES^2, bioengineered enrichment tools for the LFA with enhanced sensitivity and specificity; SEM, scanning electron microscope; N protein, nucleocapsid protein.

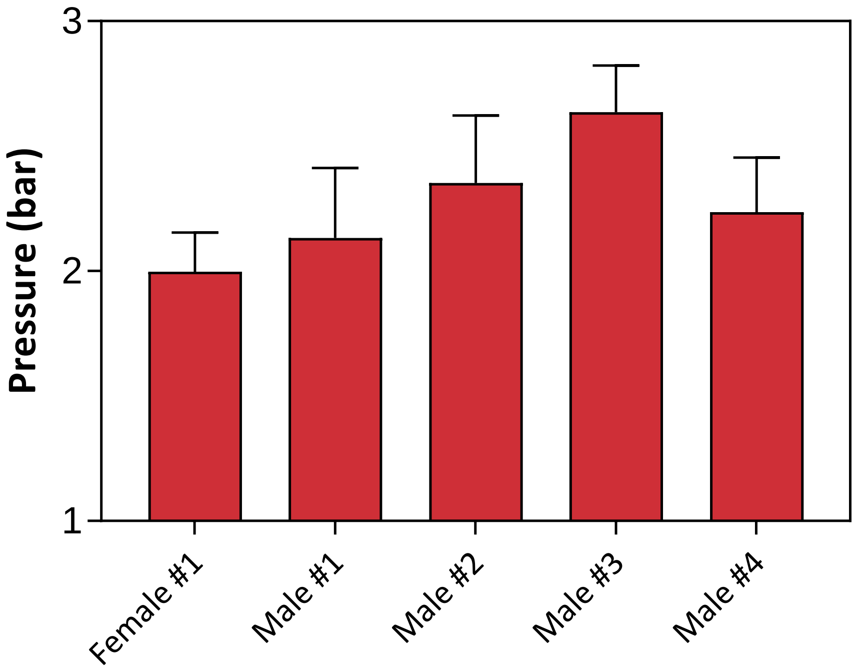

Supplementary Fig. 14. Average hand-powered pressure of 2.3 ± 0.2 bar for 3 min using the BEETLES^2 device from five individuals (4 men and 1 woman). Error bars represent standard deviation from the mean. BEETLES^2, bioengineered enrichment tools for the LFA with enhanced sensitivity and specificity.

Supplementary Table 1. Performance characteristics of the commercial COVID-19 Ag LFA kit (Sensitivity: 97.5 %; Specificity: 100 %). Ag, antigen; LFA, lateral flow assay.

Clinical performance Result		RT-PCR method		Total
		Positive	Negative	
**ALLCheck COVID-19 Ag**	Positive	78	0	78
	Negative	2	60	62
**Total**		80	60	140

Supplementary Table 2. Summary of sample isolation and enrichment techniques with their properties, advantages, limitations, target virus, figures of merits (FOM), and analysis time.

Isolation and enrichment technique	Properties	Advantages	Limitations	Target virus	Figures of merits (FOM)	Analysis time (min)	Reference
Microbead	Charge	- Short operating time<br>- Not demanding external instruments	- Low throughput<br>- Demanding operating steps	***SARS-CoV-2***	Recovery rate: 33.9±13.8%	Sample prep. 30 min	1
	Immuno-affinity			Influenza A	Recovery rate: 90%	Sample prep. 35 min	2
	Immuno-affinity			Influenza A	Recovery rate: 50%	Sample prep. 25 min	3
Centrifugation	Density	- High throughput<br>- Simple operation steps	- Demanding external instruments<br>- Poor isolation resolution (unable to discern similar density particles)	***SARS-CoV-2***	Recovery rate: 69%	Sample prep. 1280 min	4
				murine hepatitis virus (MHV)	Recovery rate: 33.5±12.1%	Sample prep. 135 min	5
Dielectrophoresis	Size and polarizability	- Low cost for device fabrication<br>- Short operating time<br>- Automation	- Demanding volume dependent force<br>- Demanding external instruments<br>- Buffer dependent performance (reduced performance in high ionic strength buffer)	T7 phage	Sensitivity: 10^4 particles/mL	Sample prep. +Analysis 5 min	6
				MS2 virus	ANOVA followed Tukey post hoc test: p-value < 0.035	Sample prep. +Analysis 1 min	7

Acoustofluidics	Size and acoustic contrast factor	- Excellent biocompatibility<br>- Precise particle manipulation (trapping, enrichment, isolation)<br>- Buffer independent performance<br>- Automation	- Demanding volume dependent force<br>- Demanding external instruments	Japanese encephalitis virus	Separation efficiency: 99%	NA	8
				Dengue virus	Extract efficiency: 90%	Sample prep.<br>9 min	9
Electrokinetics	Size and charge	- Simple operation<br>- High throughput	- Buffer dependent performance (reduced performance in high ionic strength buffer)	Baculovirus (AcNPV)	Concentration rate: 1.2	NA	10
Membrane filtration	Size	- Hand actuated<br>- Short operating time<br>- Commercially available	- Clogging<br>- Poor isolation resolution (unable to discern similar size particles)	Influenza A	virus capture efficiency:<br>96.5±0.5%	Sample prep.<br>1 hour	11
				Hepatitis C virus	Enrichment efficiency: 91%	NA	12
		- Automation<br>- Short operating time	- Demanding external instruments<br>- Enrichment capability only for intact viruses<br>-Limited in commercial inactivated lysate buffers (i.e., VTM)	***SARS-CoV-2***	Preconcentration: 40-folds<br>No Ct value increase for lysed virus	Sample prep.<br>3 min<br>+<br>Analysis<br><17 min	13
	Size and charge	- Charge-based isolation and enrichments (permselectivity)<br>- Size-based isolation and enrichments<br>-Powerful enrichment capability for both intact viruses and their N proteins<br>- Short operating time<br>- Hand actuated	- A leak-free design is needed.<br>- Volume issue in serological rapid kit.	***SARS-CoV-2 (Delta, omicron)***<br>&<br>Influenza A/B	Preconcentration: up to 34.3-folds<br>Ct value increase up to 5.1	Sample prep.<br>3 min<br>+<br>Analysis<br>15 min	***This works***

Supplementary References

1. Lázaro-Perona F, *et al.* Evaluation of two automated low-cost RNA extraction protocols for SARS-CoV-2 detection. *PLoS One* **16**, e0246302 (2021).

2. Bai Z, *et al.* Rapid Enrichment and Ultrasensitive Detection of Influenza A Virus in Human Specimen using Magnetic Quantum Dot Nanobeads Based Test Strips. *Sensors Actuators B: Chem* **325**, 128780 (2020).

3. Li G, *et al.* Influenza Virus Precision Diagnosis and Continuous Purification Enabled by Neuraminidase-Resistant Glycopolymer-Coated Microbeads. *ACS Applied Materials & Interfaces* **13**, 46260-46269 (2021).

4. Gias E, Nielsen SU, Morgan LAF, Toms GL. Purification of human respiratory syncytial virus by ultracentrifugation in iodixanol density gradient. *J Virol Methods* **147**, 328-332 (2008).

5. Ahmed W, *et al.* Comparison of virus concentration methods for the RT-qPCR-based recovery of murine hepatitis virus, a surrogate for SARS-CoV-2 from untreated wastewater. *Science of The Total Environment* **739**, 139960 (2020).

6. Yeo W-H, Lee H-B, Kim J-H, Lee K-H, Chung J-H. Nanotip analysis for dielectrophoretic concentration of nanosized viral particles. *Nanotechnology* **24**, 185502 (2013).

7. Han C-H, Woo SY, Bhardwaj J, Sharma A, Jang J. Rapid and selective concentration of bacteria, viruses, and proteins using alternating current signal superimposition on two coplanar electrodes. *Sci Rep* **8**, 14942 (2018).

8. Liu Z, *et al.* Fluorescent labeling based acoustofluidic screening of Japanese encephalitis virus. *Sensors Actuators B: Chem* **322**, 128649 (2020).

9. Fong EJ, *et al.* Acoustic focusing with engineered node locations for high-performance microfluidic particle separation. *Analyst* **139**, 1192-1200 (2014).

10. Mogi K, Hayashida KEI, Honda A, Yamamoto T. Development of Virus Concentration Device by Controlling Ion Depletion Zone for Ultrasensitive Virus Sensing. *Electronics and Communications in Japan* **100**, 56-63 (2017).

11. Yeh Y-T, *et al.* Tunable and label-free virus enrichment for ultrasensitive virus detection using carbon nanotube arrays. *Science Advances* **2**, e1601026 (2016).

12. Jeon G, Jee M, Yang SY, Lee B-y, Jang SK, Kim JK. Hierarchically Self-Organized Monolithic Nanoporous Membrane for Excellent Virus Enrichment. *ACS Applied Materials & Interfaces* **6**, 1200-1206 (2014).

13. Chen Y, Liu F, Lee LP. Quantitative and ultrasensitive in situ immunoassay technology for SARS-CoV-2 detection in saliva. *Science Advances* **8**, eabn3481 (2022).
