# Supplementary material for: PCR-like performance of rapid test with permselective tunable nanotrap
Source: Nat Commun. 2023 Mar 18;14:1520. doi: 10.1038/s41467-023-37018-6 (PMC10024276; doi:10.1038/s41467-023-37018-6)
Supplement: Supplementary file 3 — Description of Additional Supplementary Files [file 41467_2023_37018_MOESM3_ESM.pdf]

Description of Additional Supplementary Files

Title: Supplementary movie

Description: The overall workflow of BEETLES[2]. Enrichment of SARS-CoV-2 N protein and commercial LFA analysis can be done in less than 20 minutes.
